# Understanding the attitudes and experiences of people living with potentially stigmatised long-term health conditions with respect to collecting and sharing health and lifestyle data

**DOI:** 10.1177/20552076221089798

**Published:** 2022-04-18

**Authors:** Richard Brown, Elizabeth Sillence, Lynne Coventry, Emma Simpson, Jo Gibbs, Shema Tariq, Abigail C. Durrant, Karen Lloyd

**Affiliations:** 1 Department of Psychology, 5995 Northumbria University, Newcastle, UK; 2 36541 The NHS Business Services Authority, Newcastle, UK; 3 Institute for Global Health, 4919 University College London, London, UK; 4 Open Lab, 5994 Newcastle University, Newcastle, UK

**Keywords:** data sharing, patient-generated data, long-term health conditions, trust, identity, privacy, security, stigma

## Abstract

**Background:**

The emerging landscape of patient-generated data (PGData) provides an opportunity to collect large quantities of information that can be used to develop our understanding of different health conditions and potentially improve the quality of life for those living with long-term health condition (LTHCs). If the potential benefits of PGData are to be realised, we need a better understanding of the psychological barriers and facilitators to the collection and beneficial sharing of health and lifestyle data. Due to the understudied role that stigma plays in sharing PGData, we explore the attitudes and experiences of those living with potentially stigmatised LTHCs with respect to collecting and sharing health and lifestyle data.

**Methods:**

This study used semi-structured interviews and a card sorting task to explore the attitudes and experiences of people living with potentially stigmatised LTHCs. Fourteen adult participants who reported having a range of conditions were recruited in England. Template analysis was used to analyse interview transcripts and descriptive statistics were used for the card sorting task.

**Results:**

The findings present four overarching themes: Preferences for collecting health and lifestyle data, Importance of anonymity, Expected use of data, and Sources of emotional support. Participants illustrated a general willingness to share health and lifestyle data; however, there were some notable differences in sharing experiences, varying both by information type and recipient group. Overall, participants did not identify health-related stigma as a barrier to collecting or sharing their personal health and lifestyle data.

**Conclusions:**

We outline a number of preferences that participants feel would encourage them to collect and share data more readily, which may be considered when developing data sharing tools for the future.

## Introduction

Recent decades have seen a steady increase in life expectancy in many high-income countries across the world.^1^ Most of the rise in life expectancy has been attributed to declining rates of mortality amongst older adults as a result of improvements in healthcare.^2^ In the coming years, it is expected that life expectancy will continue to increase in industrialised nations, raising growing concerns surrounding the impact on healthcare.^3^ Increase in life expectancy does not necessarily translate into health improvements overall, prompting interest in the study of long-term health conditions (LTHCs).^4^ In England, as many as 20 million people are currently living with an LTHC, defined by the Department of Health in England as “*one that cannot currently be cured but can be controlled with the use of medication and/or other therapies.*”^5,6^ It is further projected that the number of individuals living with four or more chronic conditions in the UK is likely to double between 2015 and 2035.^7^ Managing the impact of LTHCs has far-reaching implications for both the individual affected and society as a whole.^6^ The demands of managing an LTHC can have a detrimental effect on an individual's quality of life.^8^ Furthermore, these demands contribute towards the growing costs of providing long-term care in the UK (both in terms of health and social care) with annual government expenditure estimated at £48.3 billion.^9^

In order to tackle the challenge of LTHCs, empowering individuals to take an active role in managing their own health is essential.^8^ Some patients report taking great value from having the opportunity to actively engage in the collection of health and lifestyle data.^10^ The ever-increasing availability of technology has enhanced the ability of people living with LTHCs to record, monitor and track their activity, symptoms and experiences.^11^ Around the world, there is a growing body of research exploring the potential benefits of using patient-generated health data (PGData) from electronic devices to improve patient outcomes in a range of conditions such as diabetes, obesity, heart disease as well as other chronic conditions.^12^ For example, in a randomised control trial of 100 heart failure patients, the use of a digital device for self-monitoring of weight, blood pressure and ECG readings was found to increase the reported quality of life through improved self-care.^13^ Furthermore, in a study of 66 metastatic breast cancer patients, 74% reported that using an electronic tablet helped them to remember symptoms, enabling them to share health information with their clinician.^14^ Increasing engagement with the collection and sharing of health and lifestyle data has the potential to improve the management of LTHCs, utilise the experiences of patients for the benefit of others as well as contribute to the understanding of different conditions.^11^ This aligns with the clear commitment of the NHS to providing a digital future in which patients become active participants in ‘citizen health’ to both benefit from and contribute to their national health service.^15,16^ For this vision of a technology-focussed health system to materialise, it is likely that patients will be required to actively collect, monitor, share and manage their health data. However, people have varying preferences with respect to collecting and sharing their own data. It is important to understand these preferences to avoid burdening individuals with unwelcome responsibility.^17^ For example, research into the perspectives of those living with multiple LTHCs found that managing PGData has the potential to become a time-consuming burden that adds to the struggles of their existing illnesses.^18^ Older adults may also struggle with PGData. Recent research has highlighted a number of barriers to the uptake of mobile-based mental health interventions among older adults.^19^ Such barriers can add to the effects of the ‘digital divide’ in which those with insufficient access, knowledge or propensity to successfully manage digital health tools may be less likely to experience positive health outcomes. The reliance on digital solutions during the COVID-19 pandemic has shone a spotlight on the digital divide,^20^ therefore, any health initiatives that look to enable the increased use of PGData must be mindful of issues of digital exclusion.^16^

Although digital health tools and services are often presented as low-cost and patient centred, many have the potential to add to the increasing workload of Healthcare Professionals (HCPs). Supporting patients to effectively share health and lifestyle data with their HCPs means considering the tools used, the data collected and the format for sharing.^21^ Increased sharing of PGData inevitably leads to increased contact with healthcare services. This is being considered at a time when HCPs are under huge pressure, exacerbated by the COVID-19 pandemic. The most frequent users of hospital services use a disproportionate amount of healthcare resources and yet their high level of received care does not always correspond with a high level of need.^22^ This pattern of use is worsened by the virtual health initiatives made necessary by COVID-19, with some patients receiving excessive levels of care while others miss out on vital services.^22,23^ In summary, it is important to ensure that supporting and encouraging the beneficial sharing of health and lifestyle data does not unnecessarily burden patients or healthcare providers. These considerations highlight the need to understand attitudes, experiences and preferences relevant to the sharing of health and lifestyle data with others.

It is suggested that in order to benefit from large quantities of PGData, people with LTHCs should be supported and encouraged to collect and share information about their health.^24^ However, despite the potential benefits of sharing PGData, a variety of obstacles prevent the widespread acceptance of these emerging data collection practices. Concerns about the security of sharing private information may prevent beneficial sharing.^25^ In the UK, the extensive disruption to the NHS that resulted from the 2017 WannaCry incident demonstrated that even large healthcare institutions are not immune to cyber-attacks.^26^ The effect of such high-profile attacks can erode public confidence in the ability of certain organisations to protect personal data.^27^ The perceived security of personal information can have special significance for those living with potentially stigmatised conditions, due to fears surrounding the disclosure of health status and concerns about confidentiality.^28,29^ A range of health conditions are associated with significant stigma,^28^ such as living with HIV,^30,31^ mental health problems,^28,32^ and chronic pain.^33^ People living with LTHCs that are typically associated with stigma may anticipate discrimination, harm or negative labels when considering whether or not to share health and lifestyle information with others.^34^ People living with LTHCs who anticipate stigma associated with their condition(s) may be less open about their experiences of health which could potentially impede them from receiving an appropriate level of care.^35,36^ Experiences of stigma can impact the way in which those living with LTHCs choose to share information. For example, previous research suggests that those who anticipate health-related stigma may seek to use online forums because they provide an accessible platform through which personal information can be disclosed anonymously.^37^ A failure to sufficiently protect the digital identities and data of those living with a stigmatised condition could leave patient identities exposed in the physical world, opening the possibility to discrimination and other harmful consequences of stigma. This study is conducted as part of a UK EPSRC funded programme (EP/R033900/2)^38^ examining trust, identity, privacy and security concerns around the sharing of self-generated health and lifestyle data primarily among people living with HIV, but also for those with other potentially stigmatised conditions. Considerations of trust, identity, privacy and security have been found to be essential when seeking to facilitate data sharing in those living with HIV.^30^ In this study, we examined whether these issues play a role in the attitudes and experiences of those living with other potentially stigmatised LTHCs.

Given the breadth of the emerging PGData landscape, and the broadly held concerns surrounding the privacy and security of health and lifestyle data, research suggests that people are eager to have control over what information is shared and with whom.^39^ Understanding individual sharing preferences may provide insights into how the collection of health and lifestyle data can be better supported and encouraged. This has particular importance for those living with potentially stigmatised LTHCs due to the influence that anticipated stigma can have on their attitudes and experiences of sharing health and lifestyle data.^40^ Previous research has considered sharing attitudes and experiences among people living with particular stigmatised health conditions (such as HIV,^30,41,42^ diabetes,^43–45^ and mental health conditions^46–48^ or has focused on a single sharing context,^18,49^ or a specific tool or platform.^45,46,50,51^ In this study, we examine the broader experience of sharing by those living with a range of potentially stigmatised LTHCs across multiple sharing contexts to gain a clearer understanding of the key overarching issues. Therefore, the aim of this study is to explore the attitudes and experiences of people living with potentially stigmatised LTHCs with respect to collecting and sharing their personal health and lifestyle data across multiple contexts. This will help to identify key psychological facilitators and barriers to the beneficial sharing of health and lifestyle data.

## Method

### Participants

This study was approved by the Department of Psychology Ethics Committee (17949) at Northumbria University. Participants were invited to participate in the study through social media (Twitter and Facebook), a local mental health charity (nondisclosed to protect patient identities), as well as advertising through the university intranet. The recruitment process invited people living with potentially stigmatised LTHCs to participate in the study. An LTHC has been defined as an incurable condition that is managed through medication or treatment.^5^ Advertising for this study invited individuals to participate who reported having one or more LTHC known to be associated with health stigma. These conditions included type 2 diabetes, mental health conditions, eating disorders and some sexually transmitted infections (e.g. Genital Herpes). However, to include those participants who anticipate health-related stigma as a result of their LTHC, but whose condition may not typically be associated with stigma, recruitment left open the possibility for individuals to self-define their own LTHC(s) as being potentially stigmatised. There were no restrictions on gender, sexual orientation or upper age limit, but participants were required to be aged 18 + and currently residing in the UK. The study recruited 14 participants (four men and 10 women, all cisgender). The mean age of participants was 42.7 years (ranging from 22 to 65) and all were white British with the exception of two participants (one Lebanese woman and one woman who declined to specify their ethnicity). Each of our sample reported one or more of the following potentially stigmatised LTHCs: arthritis, chronic obstructive pulmonary disease, complex post-traumatic stress disorder, coronary heart disease, depression, emotionally unstable personality disorder, general anxiety disorder, genital herpes, inflammatory bowel disease, mental health conditions, myalgic encephalomyelitis, thyroid disease, and type 2 diabetes.

### Data collection

#### Interviews

All participants took part in semi-structured interviews, conducted using a schedule designed to explore participant attitudes and experiences relevant to collecting and sharing health and lifestyle data. Prior to the interview, participants were given an information sheet to enable them to provide informed consent. The researcher asked participants about their preferences for collecting information about their health and lifestyle and their attitudes and experiences of sharing this information with others. Participants were asked about what, how and why they collect information about their health and lifestyle. Interviews then addressed participants’ contact with health services by asking what and how they share information with HCPs and their attitudes towards doing so. Interviews also explored attitudes and experiences of sharing health and lifestyle information with a broad range of recipients, such as charities, peer support groups, family, friends and work. Participants were asked about how they would feel about their personal health and lifestyle information being shared with other groups, such as with pharmaceutical companies, academic research, health charities, or other commercially interested organisations. Finally, interviews asked participants about their experiences of health-related stigma with respect to their attitudes and experiences of sharing with others. Twelve interviews were conducted face-to-face, one was conducted via Skype and one was completed via email exchange. Interviews lasted between 33 and 90 min with an average interview time of 58 min. Interviews were digitally recorded and subsequently transcribed verbatim. During the transcription process, all identifying information was pseudonymised with participants subsequently being referred to by participant number.

#### Card sorting task

Following the interview, participants were asked to participate in a modified version of a comfort card sorting task.^30^ This task provides a visual analogue scale, similar to those used in previous health research to allow participants to provide a comparable measure of their attitudes and experiences.^30,52^ This method was used in addition to the semi-structured interviews to provide additional means through which participants could express and rate their attitudes towards sharing different categories of health and lifestyle information with a range of recipient groups. Participants were presented with cards containing a single piece of personal, health or lifestyle information (total *n* = 27). Participants were also presented with seven different recipient groups: HCPs, public health/research, other people with a similar condition, family, friends, work and social media. Participants were asked to sort each of the information types into ‘*Yes, willing to share*’, ‘*Unsure’* or ‘*No, unwilling to share*’ for each recipient group and were asked to think aloud so that explanations could be captured. Due to missing data and some participants not completing the task, card sorting task data were collected for 11/14 participants (all excluding participants 4, 5 and 14).

### Data analysis

#### Interviews

The participants’ responses were assessed by conducting a Template Analysis of the transcripts. Following a Template Analysis approach allows the researcher to identify and define *a priori* themes that depict topics and concepts that are of interest and relevance to the study.^53,54^ Template Analysis has much in common with other forms of thematic analysis, most notably Framework Analysis.^53^ However, although these approaches can be implemented to meet many of the same research needs, the most notable difference is that Template Analysis provides a more detailed development of the coding structure.^53^ This method was chosen to help organise the breadth of experiences of those living with potentially stigmatised LTHCs with respect to the *a priori* themes dictated by the research program (see below). Furthermore, Framework Analysis may be considered more theoretically focussed and most suited to applied policy research,^55^ whereas Template Analysis is often used in a wider variety of research settings and is now well embedded in qualitative healthcare research.^56^ This chosen technique allowed for the initial analysis to focus on developing concepts that have been previously associated with health information sharing behaviours, such as trust, identity, privacy and security.^39,57,58^ Throughout the analysis, key themes were expanded, or even discarded if they were found not to accurately portray the participants’ responses. This allowed for additional themes to come to light and for a more diverse range of ideas to represent the overall participant account.

The process of developing the initial template began by reviewing the content of each transcript. A preliminary thematic coding of the transcripts was conducted in alignment with *a priori* themes, whilst allowing the increasing familiarity with the transcripts to guide the addition of new themes. Clusters of new themes and topics of interest were further analysed to produce superordinate themes. These were used to construct the initial template. This initial template was then applied to the interview data and subsequently modified to consolidate the themes and structure. This was discussed amongst all members of the research team to ensure that the resulting template reflected a collective analysis of the participants’ responses. The final version of the coding template led to the selection of four themes representing the participants’ experiences and attitudes towards the collection and sharing of health and lifestyle data. Verbatim extracts from the transcripts are presented and discussed below to illustrate the findings.

#### Card sorting task data

The participant responses to the card sorting task were coded numerically (‘*Yes, willing to share*’ = 3, ‘*Unsure*’ = 2, ‘*No, unwilling to share*’ = 1) to provide a score for each combination of information type and recipient group. The data for each of the 11/14 participants that completed the task were then combined to provide an analysis of the overall sample's sharing preferences with respect to information type, recipient group and each combination of the two.

## Results

### Card sorting task data

 Table 1 presents the combined sharing preferences of our sample for each combination of information type with recipient group. Overall, participants indicated that they were broadly willing to share most information types with most recipient groups.

**Table 1.** Combined responses for card sorting task.

	Healthcare professionals	Public Health & research	Other people with the condition	Family	Friends	Work	Social media
**Name**	33	32	32	32	32	33	32
**Age**	33	33	32	32	32	31	30
**Photo of myself**	30	24	27	32	32	31	28
**Relationship Status**	32	32	33	31	32	32	32
**Ethnicity**	32	32	32	32	32	31	28
**Gender**	33	32	33	32	32	32	29
**Email Add**	32	28	29	32	32	31	26
**Area I Live**	33	32	29	32	32	33	31
**Family Status**	32	32	33	32	32	32	27
**Date of diagnosis**	33	32	30	31	31	32	25
**Sexual Orientation**	27	28	27	27	29	28	22
**Medication side effects**	30	31	31	30	29	28	23
**Medication**	33	33	31	31	31	30	25
**Symptoms**	33	33	33	32	32	31	26
**Emotion**	32	33	33	32	32	31	29
**Mood**	32	33	33	32	32	32	30
**Weight/BMI**	31	33	33	30	32	29	27
**Exercise**	32	33	32	32	31	32	31
**Sleep**	33	33	33	32	31	33	32
**Condition management**	33	33	33	32	32	30	23
**Diet/nutrition**	33	33	33	32	32	31	28
**Other Health concerns**	33	33	24	32	32	31	24
**Struggles**	31	33	30	28	29	29	26
**Substance use**	30	33	33	30	32	28	24
**Sexual health**	27	31	28	27	27	24	23
**Sexual activity**	23	25	23	22	25	22	18
**Mental Health**	32	31	31	30	32	30	27

*Note.* For each combination of information type and recipient group there is a minimum score of 11 (all participants unwilling to share) and a maximum of 33 (all participants willing to share).

**Table:** 

*Traffic light coding for combined scores*							
11–12	13–15	16–18	19–21	22–24	25–27	28–30	31–33
							
Everyone unwilling to share			Unsure about sharing				Everyone willing to share

**Figure 1.**
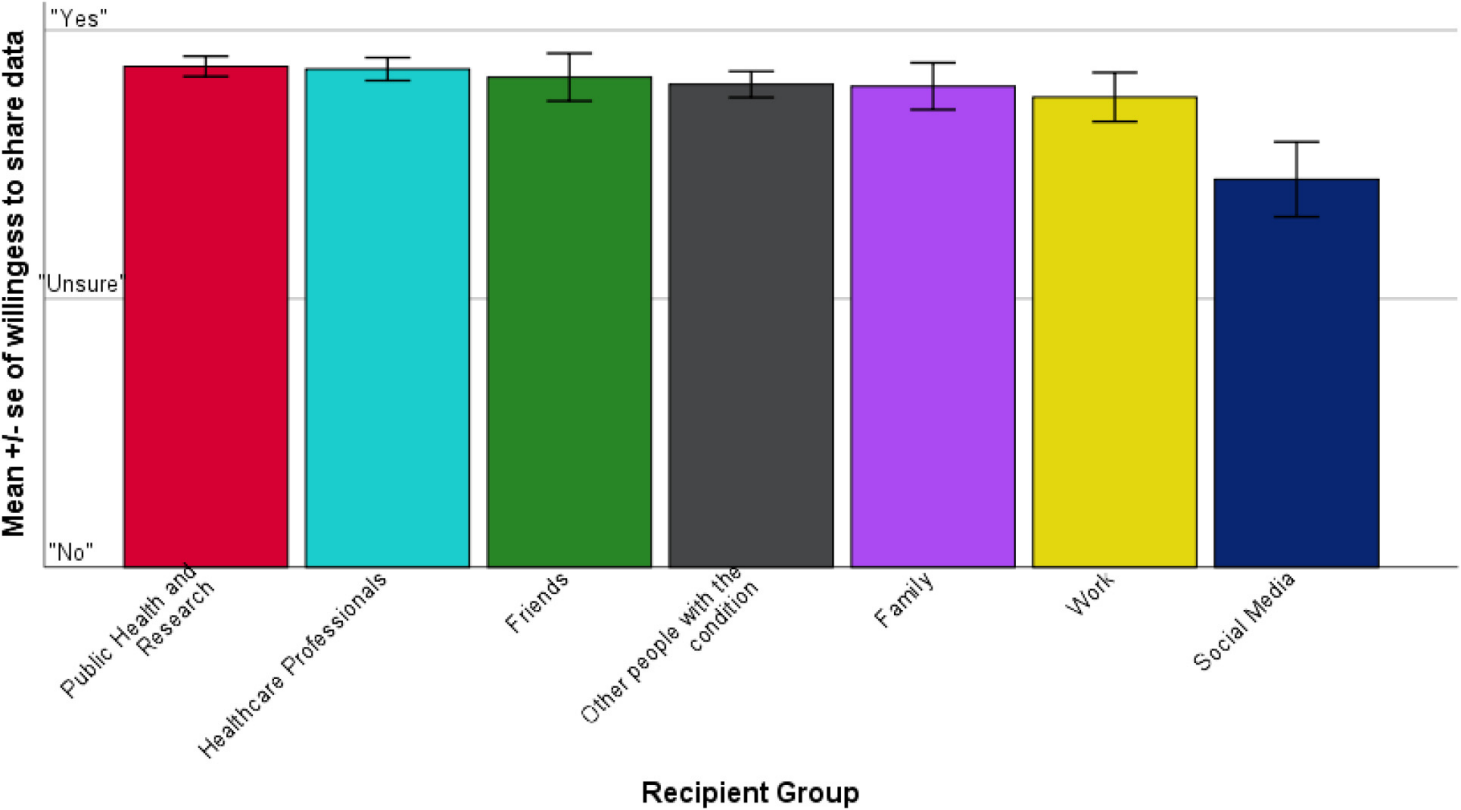
Mean participant willingness to share information by recipient group.

Figure 1 presents the mean scores for participant willingness to share by recipient group. Our sample reported being most willing to share personal information with public health and research (*M* = 2.87, *SD* = .13), and least willing to share via social media (*M* = 2.44, *SD* = .46).

**Figure 2.**
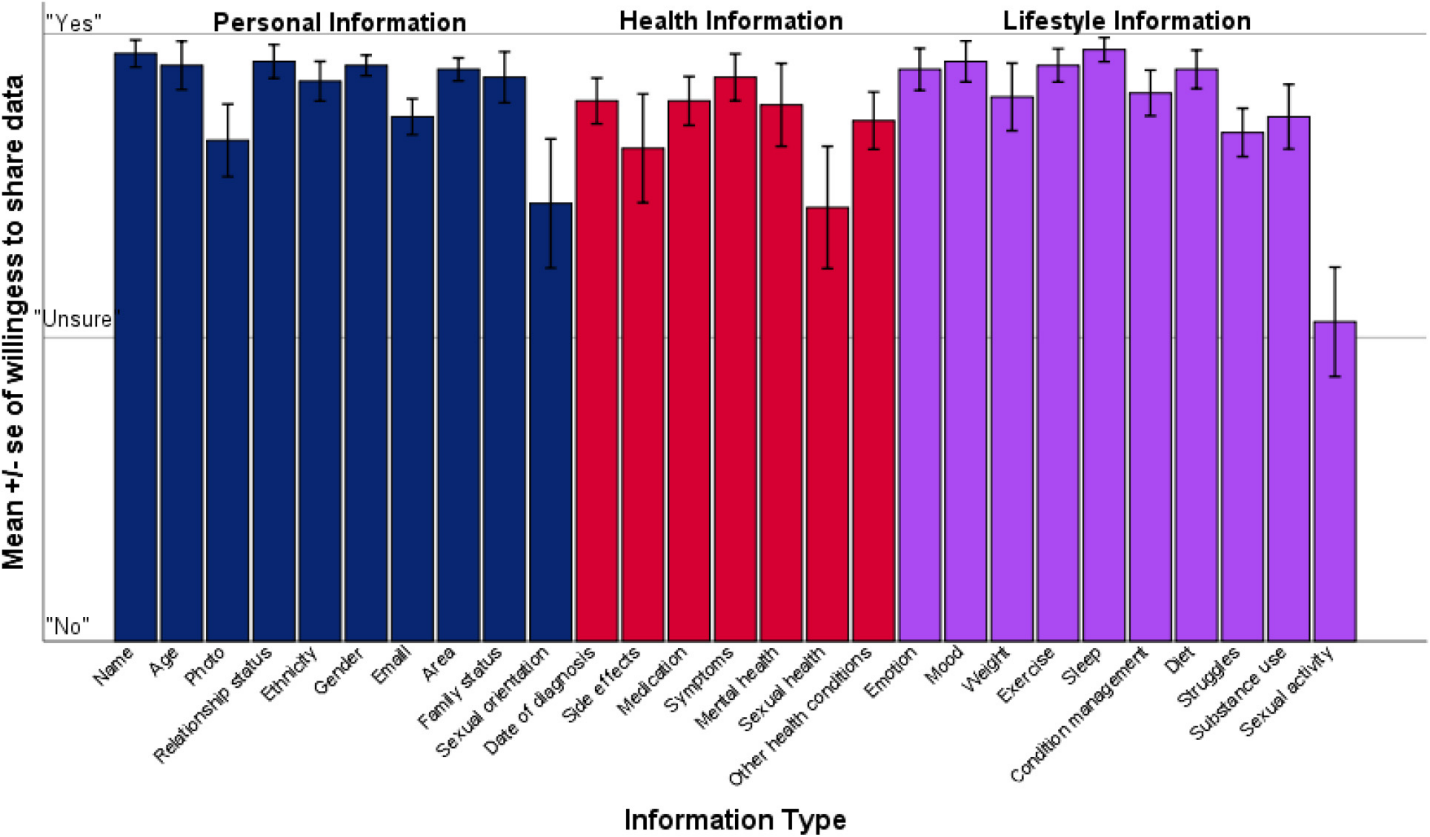
Mean participant willingness to share information by information type. *Note.* Information types are categorised into Blue = Personal data, Red = Medical data, and Purple = Lifestyle data.

 Figure 2 presents the mean scores for participant willingness to share by information type. Our sample reported being most willing to share information pertaining to sleep (*M* = 2.95, *SD* = .13), as well as their own names (*M* = 2.94, *SD* = .15), and least willing to share information about sexual orientation (*M* = 2.44, *SD* = .70), sexual health (*M* = 2.43, *SD* = .67) and sexual activity (*M* = 2.05, *SD* = .60).

A repeated-measures ANOVA determined that mean sharing scores differed between recipient groups, *F*(2.46, 24.66) = 4.44, *p* = 0.02, and between information types, *F*(3.29, 32.85) = 4.67, *p* = 0.01. However, post hoc pairwise comparisons using the Bonferroni correction showed no significant differences in mean sharing scores across recipient groups or information types. This contradictory finding between the significant global effects and the non-significant pairwise comparisons is most likely explained by a lack of statistical power due to the limited sample of 11 participants.

### Interview transcripts

In this section, we report the findings of our interview study by presenting the four themes that were highlighted from our template analysis as best expressing the overall narrative of the transcripts: Preferences for collecting health and lifestyle data, Importance of anonymity, Expected use of data, and Sources of emotional support. An overview of each theme, accompanied by a representative example from the transcripts, is presented in Table 2. Each theme is presented, defined and explored in detail below.

**Table 2.** Definition of themes and examples.

Theme title	Definition	Example quotation
1. Preferences for collecting health and lifestyle data	The extent to which collection preferences influence willingness to manage and share data.	*“Certainly that is what is missing in the trackers I have seen, so you can put in your own symptoms so you can make it relevant to you if it then has a way of identifying patterns.”*
2. The importance of anonymity	The extent to which participants expressed the importance of controlling their anonymity when considering whether to share health and lifestyle information.	“*I felt like it was an anonymous space that I could just get my thoughts completely out and I didn't have to hold back.*”
3. The expected use of data	The influence that the expected use and treatment of personal data has on an individual's willingness to share information, and how this may vary when considering different recipient groups.	*“I would not be happy with sharing any data to be used as a profiting mechanism.”*
4. Sources of emotional support	The different sources of support available and the extent to which perceived sensitivity of information, and recipient reaction, impact willingness to share.	*“I tend to share more with friends than family. It's a lot easier to share with people who you know will understand.”*

#### Preferences for collecting health and lifestyle data

Participants described their experiences of different methods used for the collection and sharing of data and discussed their desired preferences for future technology design. Seven participants reported using traditional (analogue) methods for recording health and lifestyle data. These participants highlighted the utility of keeping a physical journal. Despite many stressing the difficulty of consistently engaging with a written journal, participants noted its utility for sharing information with HCPs.

*“I started keeping a diary of the side effects of the medicine as well as any joint pain so that when I go in to see the nurse I would remember to tell her my experiences since at first I would feel so overwhelmed and emotional that I would forget to tell her all the details and ask her all the questions I had.” [Participant 14]*

This highlights the utility of basic methods of data collection and how they can facilitate healthcare interactions. Participants also described using more creative handwritten methods, either to track their feelings and experiences or as a form of catharsis and expression.

*“There’s one thing I do track. I, kind of, track my moods… What I have is I’ve got a… It’s not in a numerical way. I do a morning doodle. I draw and write what’s in my mind every morning, and I’ve got a reflective journal. I do a gratitude journal, so I do all of that.” [Participant 11]*

*“Because I’m quite visual I’ll often draw smilies or just doodles, which reflect the day as I’ve experienced it.” [Participant 1]*

In contrast, eight participants reported using digital devices accompanied by a range of applications to record, track and monitor symptoms and other health-related information.

*“With my medications I have had some heart rate issues so this also has the heart rate tracker, so I can watch that to make sure it’s not trying to go way high, it’ll drop sometimes. I use just the health app on my iPhone to track my cycles and symptoms involved with that, and trying to figure out when exactly things are going to start, and when I can expect to feel the worst” [Participant 13*

Participants described using digital devices to collect a range of health-related data and that this process helped them to self-manage their condition(s). Although the perceived utility of digitally recording and sharing data varied by participant, many stated that they would be more willing to use technology if certain preferences were met. For instance, roughly one-third of participants indicated that the ability to combine traditional handwritten methods with the use of digital devices would make data recording and sharing more convenient.

*“I have an iPad that has an app that allows me to hand write into it, I use an Apple Pencil, and I find that I retain the information better if I can write it out by hand versus than type it. And I am horrible at typing, so you know this is kind of the best of both worlds.” [Participant 13]*

*“That’s kind of what the bullet journal method says, it’s about analogue tools are still valid in a digital world.” [Participant 1]*

In addition to incorporating analogue methods into a digital platform, participants also expressed a desire to combine multiple existing applications for added convenience.

*“You find you can get very, very specific apps and you can just end up swamped with apps… Something that would be more streamlined and in principle something that could bring things together would be really useful.” [Participant 1]*

*“Some sort of marriage between physical tracking but also being able to track you know major symptoms would be very very helpful. And if there is an app out there that does that, then I haven’t found it, and I have probably gone through just about every app possible.” [Participant 13]*

The advantage of merging different applications was described as being to combine existing sources of information which could provide fresh insight into the interplay of various health and lifestyle factors. Participants highlighted this as being a key goal for using technology to better understand their condition and how best to manage it.

*“Certainly that is what is missing in the trackers I have seen so you can put in your own symptoms so you can make it relevant to you if it then has a way of identifying patterns, have you noticed that two days after this happens you crash or if you eat an avocado you feel better.” [Participant 8]*

*“So, if I could map my activities with my symptoms, I would find that really useful, or if I’ve spent a day in the office looking at the computer screen and I haven’t moved very much, how does that impact my next day, so I could better manage things.”* *[Participant 5]*

Overall, participants highlighted a need to be able to combine traditional methods of data collection and expression with existing applications in a convenient and integrated way. Participants described their motivations for using such technologies in terms of being able to better understand the interaction between different health and lifestyle factors so that they could improve the self-management of their condition.

#### The importance of anonymity

This theme refers to the extent to which participants expressed the importance of controlling their anonymity when considering whether or not to share personal health and lifestyle information. Almost all participants identified the ability to remain anonymous as an enabler of sharing. Online health message boards were highlighted as a platform on which anonymous sharing can take place.

*“I shared quite a lot of information. In fact, I probably overshared. I felt like it was an anonymous space that I could just get my thoughts completely out and I didn't have to hold back in any way because it's a group of people who would already understand what I'm going through.” [Participant 12]*

The ability to remain anonymous was described as enabling uninhibited sharing without being concerned about the impact of expressing one’s experiences. Ensuring that an individual's anonymity could be protected was also reported as enabling the sharing of personal information with recipient groups that were otherwise distrusted, such as pharmaceutical companies, advertising firms and other commercial interests.

*“In terms of advertising, I think it's something that I don't have any control over anything at the moment, so that's already going on. As long as I was anonymous … So, if it's in terms of pharmaceutical companies, as long as I was kept anonymous as a person, that wouldn't bother me so much.” [Participant 12]*

Although 11/14 participants expressed concerns about the perceived practices of commercially interested companies, anonymity acted as an enabler of sharing, including with third parties. Despite distrusting or disapproving of certain industry practices, guaranteeing that one's name would not be ascribed to the data appeared to mitigate some concerns about sharing.

The importance of anonymity was also expressed in relation to concerns about shared data being linked back to an individual's ‘real life’.

*“My concern would be more about people in real life seeing it than people who were just online. I suppose my other concern about online is how findable I am. If I did put something and I had all the privacy settings in the world… Having my double-barrelled surname and what I do… My clients will search for me.” [Participant 11]*

Participants were concerned about information that is not shared anonymously being available to those in their private, social or professional lives. Even in cases where an individual's name may accompany shared data, so long as the participant felt that this would remain distinct from their ‘real life’, a sense of anonymity could still be maintained.

*“If it is for research, I am assuming it is not yourself, not someone I know, that I am then disclosing all my stuff to, then it is anonymous, even though its got your name attached to it they don’t know you so I would feel anonymous so I would be quite happy to share all of them.” [Participant 8]*

When discussing the possibility and effects of potential breaches of anonymity, half of all participants expressed significant concerns. However, there was uncertainty as to what participants could do in response to such a breach.

*“Oh yes, I'd be livid. I mean, if I found out about it, I'd be bloody livid, that's for sure. I don't know what you do about it these days, though. I suppose that's the thing.” [Participant 2]*

Despite almost all participants expressing a need for anonymity, those who expressed concerns suggested that they may not be able to control whether their data remains anonymous. To avoid the potential for broken guarantees of anonymity, there was a broad desire for the ability to limit the initial sharing of identifiable information with certain recipients so that the individual may have a greater sense of agency and control.

*“You know in the idea of sharing more information, if there was a way to strip identifying information out, I would not be afraid to put out more information. Its having that like even on PatientsLikeMe I don't put my name on things, a lot of things I just sign with initial, there is still enough linked to me that it is scary” [Participant 13]*

This desire to choose to remain anonymous when disclosing certain health data was again described as having the potential to enable sharing. Whether or not participants trusted that their data would remain anonymous depended on the degree of control they perceived they had not to disclose identifying information, as well as the expected use and treatment of data.

#### The expected use of data

This theme addresses the influence that the expected use and treatment of personal data has on an individual's willingness to share information, and how this might vary when considering different recipient groups. When asked to consider with which recipient groups participants would be willing to share, responses were driven by the perceived purpose for the collection of data.

*“…again, I think the big question would be ‘What’s the purpose? ‘What are you going to do with it?’” [Participant 7]*

In addressing the expected use of data by recipient group, most judgments were based on whether or not the intended purpose for the use of information was perceived to be in the public interest.

*“I wouldn’t mind that, because it’s all going towards future research, and a lot of people with chronic health conditions are very, very happy to help, if it’s going to help somebody else.” [Participant 6]*

*“I think it depends on what the purpose is. If it’s for research and helping people, or helping medical knowledge or something like that, then that’s fine by me. But if it’s for making money or making an app better or something, then I would have to consider that in more detail. It really depends on the person. Charity I’m more fine with, again, because it’s not for making money.” [Participant 4]*

Recipient groups that were deemed to be in the public interest included universities and other higher education institutions, as well as public health organisations such as Public Health England. Public interest groups were discussed in stark contrast to commercially interested organisations, most notably pharmaceutical and advertising companies.

*“I would not be happy with sharing any data to be used as a profiting mechanism. Accessing health services and treatments is a public right and as such I am against the profiting of private companies within this space.” [Participant 14]*

This general reluctance to share personal information with commercially interested organisations was not discussed in terms of the specific industry practices, but rather in terms of a broadly held belief that organisations should not be permitted to profit from the sharing of personal health information, and that organisations that seek to do so should not be trusted.

There were further questions of trust with respect to the expected treatment of shared data, and whether this treatment of data could guarantee the security of personal information.

*“It's not a question of not trusting them; it's not trusting their system. … Yes. Yes, if I was doing something bloody stupid and I was talking to my doctor, fine, but if you're typing a note into the system, then there's a possibility that system could go under. So that was the only one from that.” [Participant 2]*

Such concerns about the ability of systems to securely house shared information were not typically addressed by reference to specific events or concerns but rather through the lens of a general lack of trust in the ability of companies to protect private data. Over half of participants expressed fears about the potential consequences of non-secure treatment of their own shared data.

*“I would be very concerned about data leaks and security features by external companies and I definitely wouldn't want my information sold in any way. That's obviously a big one.” [Participant 12]*

*“I’d feel extremely vulnerable just on an instinctive level not knowing what that could be used for. What agenda the person had for doing that, you just don’t know. It’s the not knowing aspect that gets me.” [Participant 1]*

Although most participants did not address specific concerns that they may have with respect to how the misuse of their data could be used against them in the event that it fell into the hands of malicious actors, improper treatment of data was perceived as a real threat. Participant vulnerability to this threat was exacerbated by uncertainties surrounding the expected use of data.

#### Sources of emotional support

This theme refers to the different sources of emotional support available and the extent to which perceived sensitivity of information, as well as the expected reaction of the recipient, impact participant willingness to share. Family was described as providing a key source of emotional support for participants. Although some participants expressed that they would be willing to share all aspects of their health and lifestyle information with certain members of their family, most participants adopted a tailored approach to family sharing, both in terms of what information they would share and with whom.

*“It depends who, my friends and family. My dad I don’t really tell much to, because he’s really Old School, and sometimes I find it a bit weird to discuss personal things with him, especially regarding sexual health and mental health. My mum, with her it’s a bit iffy right now, because we don’t really have much of a relationship anymore. But I would in the past, but not now.” [Participant 4]*

*“Say, so, like, my dad, I wouldn’t share my sex life or my sexual orientation, because I think he’d have a heart attack. Substance use, no, other… he knows I drink alcohol, struggles. So, actually, substance use, I wouldn’t share with my mum because I use cannabis oil to manage my symptoms. I know she’s really against and, obviously, it’s illegal, but it’s the only thing that works sometimes, so I’m going to use it, but I wouldn’t share.” [Participant 5]*

The perceived sensitivity of the information type clearly influenced the willingness of participants to share with certain family members. This particularly applied to information of a sexual nature, information pertaining to substance use and, in some instances, mental health. Two participants described generational factors, or family members being ‘*Old School*’, as determining whether or not they would feel comfortable sharing sensitive information with family. In contrast, participants generally reported being willing to open up more readily to friends when discussing sensitive topics.

*“I think most of my friends, if they wanted to know something, I don’t hide many things and I quite openly talk about sex life and stuff with my close friends. So, yes, I don’t think there’s anything I wouldn’t share.” [Participant 5]*

*“I tend to share more with friends than family. It’s a lot easier to share with people who you know will understand or be able to empathise regardless of how well you know them, I’ve found. I check in with a lot of friends.” [Participant 1]*

This openness with friends was described in relation to their expected reaction being one of empathy and understanding. Generally, this expectation enabled a willingness to share with friends, most notably with respect to sensitive information. In contrast, the expected reaction of certain family members was described as a barrier to sharing by just under half of the participants in instances where it was felt that sharing may have negative consequences.

*“Obviously you do talk to your family and stuff about a lot of things, but I think sometimes you don’t want to say too much that’s going to start to have an impact on their mental health as well. [Participant 6]*

*“It’s opening up enough, without completely exposing or getting people to worry. My mother, bless her, she… I think I was talking to her on Facebook. She was like, ‘You’re not going to worry me, are you?’ Which is kind of fine but is also a little bit like, actually, I need to package it up in a way that she can cope.” [Participant 11]*

These excerpts illuminate that individuals may sometimes weigh up the benefits of sharing against the negative consequences for those around them. As an alternative to seeking emotional support by sharing experiences with family and friends, just under a third of participants (4/14) highlighted the value of opening up to online communities.

*“I think it’s because it’s strangers on Twitter and Instagram a lot of the time, so they don’t know you and won’t try to chase you up about if you’re okay. Sometimes you don’t want that. You just want to let it go and express yourself, I guess.” [Participant 4]*

This form of sharing provides a seemingly consequence-free form of emotional support in which an individual can share their experiences without being concerned about the reaction of those closest to them. Sharing with online communities of those who have a similar condition was also described as offering an additional form of support in the form of providing validation for the participant’s reported experiences.

*“There is a Facebook group that is actually really useful in terms of just some things that you might not even think are part of it, people saying yeah I get that, I get that as well and again more from a validation point of view.” [Participant 8]*

Those who reported engaging with groups online who may have had similar health experiences to the individual (4/14) described a form of emotional support that may not be available from family and friends. These participants highlighted the value of receiving a sense of validation from those who have had similar experiences. Online communities also provide a source of support external to an individual's ‘real life’ with which someone may share their experiences more openly without being concerned about harming those around them.

Finally, it is noted that only one participant chose to highlight the role that health-related stigma plays in their experiences of collecting and sharing health data. This participant reported living with Genital Herpes and described how their anticipation of health-related stigma had a detrimental effect on their willingness to share health and lifestyle data with most recipient groups, other than HCPs. This was seen to limit the channels of ‘emotional support’ available to the participant due to the anticipated reactions of others (including friends, family and those closest to the participant). However, the remaining participants did not identify stigma associated with their LTHC(s) as a barrier to collecting or sharing health and lifestyle data with others.

## Discussion

This study investigated the attitudes and experiences of collecting and sharing personal health and lifestyle information among those living with potentially stigmatised LTHCs. The findings of this study illustrate a general willingness to share most forms of health and lifestyle information with most recipient groups. This was typically described as being motivated by a desire to improve the self-management of the participant's own condition as well as to help others. That said, there were some noticeable differences in reported sharing experiences, which varied both by information type and by recipient group. Most notably, participants reported being less willing to share health and lifestyle data via social media than with other recipient groups. There was also a general distrust towards sharing data with commercially interested parties, in stark contrast with the broadly held willingness to share with organisations that were deemed to be in the public interest. Although individual participant preferences varied by information type, participants were less willing to share data that they deemed to be sensitive, which was particularly true for information of a sexual nature. Finally, despite the fact that all participants reported living with a potentially stigmatised LTHC, the majority of participants did not identify health-related stigma as a barrier to collecting or sharing their personal health and lifestyle data.

Across the sample, participants expressed the importance of being able to choose what information is shared, and with whom. This finding is consistent with previous research that suggests that patients desire ‘granular’ control over their health information so that they can actively select what and how it is shared.^39,59^ This need for control was also expressed by the importance that participants placed on having control over their anonymity. The ability to remain anonymous was shown to enable enhanced sharing, particularly when engaging with online communities. Anonymity has previously been suggested to encourage the sharing of health information online.^37^ In the current study, the importance of anonymity was often discussed in terms of preventing being identified by those around them. This seems to suggest that remaining anonymous in online communities can facilitate uninhibited sharing, in part due to the perceived distinction between online engagement and ‘real life’. This speaks to the utility of anonymity when managing the boundaries that individuals establish between their separate environments.^60^ The participants’ emphasis on the importance of anonymity did not translate into an absolute unwillingness to disclose personally identifying information. This is supported by participants’ own names being ranked second highest on the card sorting task in terms of overall willingness to share with others. It is suggested that the participants’ concept of anonymity does not simply refer to whether identifying information is disclosed or not, but also addresses the context in which an individual may be identified by others, as well as the degree of control they feel they have over how this is managed. This relates to previous research which has argued that decisions around privacy are not binary, instead they involve nuanced consideration of how personal information is likely to be used, by whom, and the sense of agency experienced by the individual.^61^ The distinction drawn by Raynes-Goldie (2010) between social and institutional privacy described the former as involving other people, often familiar to the individual.^62,63^ The importance of anonymity described by participants in preventing being identified in one's ‘real life’ raises concerns about social privacy, whereas the importance placed on anonymity when considering data falling into the hands of commercially interested organisations raises institutional privacy concerns. A more detailed exploration of the contextual drivers of privacy concerns in those living with potentially stigmatised LTHCs may further explain how anonymity acts as a key facilitator for the sharing of health and lifestyle data.

Where the purpose of the use of participant information was perceived as being aligned with a broader public interest, all participants were particularly willing to share. Public health and research was the recipient group with whom our sample was most willing to share. This was generally explained by participants’ motivation for their own information to be used to benefit others. Almost all participants drew a sharp line between their willingness to share with ‘public interest’ groups and ‘commercial interest’ groups. This was clearest in the overwhelming aversion to sharing personal information with pharmaceutical companies which may be indicative of an ongoing ‘trust crisis’ within the pharmaceutical industry in general.^64^ Recent concerns over the proposed central NHS digital database drawing on data from GP records in England indicates ongoing concerns regarding trust, transparency and the potential misuse of patient data.^65–67^

In addition to the impact that anticipated use of information had on a participant's willingness to share, the expected reaction of recipients strongly influenced attitudes towards sharing. This was most apparent when weighing up whether or not to share certain categories of sensitive information with family, friends or other people with a similar condition. Despite there being no clear consensus about what constitutes sensitive information, previous research has suggested five categories of sensitive health data: sexually transmitted infections, HIV/AIDS status, sexual health and pregnancy, mental health information, and substance use.^47^ Several participants discussed both sexual health and substance use as particularly sensitive and the three least shared information types from the card sorting task all related to sex. There were often clear differences in participants’ willingness to disclose sensitive information with family compared to friends. Some participants explained this by suggesting that sharing with friends tended to accompany a greater expectation of understanding, especially with respect to sensitive information and may explain why the results of the card sorting task show a slight preference to share with friends over family. The reluctance of some participants to share with family was further explained by concerns that sharing information with certain family members may provoke a negative reaction, either by causing distress to the recipient or by prompting them to take unwanted action. Again, this relates to the importance of managing boundaries between different environments. In addition to the discussed role that anonymity plays in allowing participants to manage boundaries, sharing with those who have a similar condition provides an opportunity to disclose experiences without expecting consequences in one's ‘real life’. Primarily, online health communities can validate an individual's experiences and provide a sense of understanding. Although not all participants expressed a willingness to engage with online communities, such groups provide a valuable alternative to sharing with family and friends.

The findings illustrated a clear desire for an agency in self-managing LTHCs and a general willingness to share health and lifestyle data with a range of sources. Despite broad interest in engaging with technology to facilitate the beneficial collection and sharing of data, participants highlighted that technology can be both a help and a hindrance. The flexibility of being able to combine traditional methods of data collection with digital storage and analysis appeared particularly attractive to participants. This desire for the integration of resources extended to wanting the ability to combine the benefits of a range of existing applications and devices. This combining of methods and devices would not only improve the ease with which information could be recorded and shared, but could also be used to detect patterns that occur across previously separated categories of data. The potential for pattern recognition and broader analysis may provide additional agency to those managing LTHCs and help them to better understand their experiences. Given the overall motivation to share information for the purpose of benefiting others, if people felt that engaging with this technology could improve the understanding of their condition, they may be more motivated to share.

The majority of our sample did not highlight health-related stigma as being central to their attitudes and experiences of collecting and sharing health data. Therefore, contrary to expectations, the experience of stigma was not emphasised in the analysis of participant responses as a key barrier to sharing. It is noted that the one participant that did stress the role that stigma plays in their reluctance to share health information reported having a sexually transmitted infection. Our findings highlighted that information pertaining to sexual health and activity is often considered to be very sensitive, which may account for the heightened sense of stigma reported by this participant. It was also expected that participants who reported having a mental health condition may also highlight the impact of stigma on their experiences. Despite increased mental health awareness in recent years,^68^ as well as a complex change in public attitudes towards a range of associated conditions,^69^ a broad body of literature suggests that both public stigma and self-stigma continue to have a detrimental effect on those suffering from mental health.^70^ That said, a third of our sample reported living with mental health conditions and yet chose not to stress the influence that stigma has on their accounts of managing their mental health. This suggests that living with a condition typically associated with stigma does not inevitably cause people to feel stigmatised by their health status. Furthermore, previous research has suggested that, where stigma is present, many labelled individuals actively resist feelings of being stigmatised while learning to manage the demands of their condition.^71^ As individuals become more accustomed to dealing with long-term illness, some may begin to view their experiences through a lens of normalcy and not associate them with existing stigmas. Overall, this suggests a more complex relationship between stigma and PGData sharing and one that warrants further investigation.

A potential limitation of this study is that participants’ initial agreement to take part required a certain level of trust in opening up to researchers about their health. Several participants reported a close familiarity with academic research due to the proximity of their own work to research activities. This may in part be due to the recruitment of participants through university channels. This initial agreement and familiarity may go some way to explaining the overall willingness to share data for the purposes of research. Future studies may seek to explore whether or not there is still a general willingness to share with public health and research organisations in participants that are not as familiar with their practices. Furthermore, previous research has found that socioeconomic variables such as income and education are often associated with levels of trust in public health and health information more generally.^72,73^ While this study did not collect the socioeconomic backgrounds of the participants, future studies may look to capture perceptions of those living with LTHCs from a range of socioeconomic backgrounds to explore the role that various demographic factors may play in forming attitudes towards sharing.

## Conclusion

This study adds to a growing body of literature that seeks to understand the experiences of those living with LTHCs. Our research has focussed on the attitudes and experiences of those living with potentially stigmatised LTHCs with respect to collecting and sharing health and lifestyle data across multiple contexts. Our findings suggest that having the option to remain anonymous prompts a willingness to share health and lifestyle data, particularly in online communities. Willingness to share is often determined by the perceived purpose for the use of data and, when sharing, people may seek emotional support from a range of sources depending on the sensitivity of the information they wish to disclose. We have outlined a number of technological preferences that participants felt would encourage them to collect and share data more readily, which may be considered when developing tools for the future. Identifying the factors that facilitate engagement with sharing technologies may help to harness large amounts of valuable data that can be used to improve the management of LTHCs. This has significance for understanding the underexplored experiences of those living with potentially stigmatised LTHCs, in order to support and encourage the beneficial sharing of health and lifestyle data.
